# The Epigenetic Fingerprint of Lifestyle: Smoking, Vaping, and Exercise Revealed Through Buccal DNA Methylation

**DOI:** 10.3390/genes17040369

**Published:** 2026-03-25

**Authors:** María Josefina Castagnola, Mayaas Hassan, Varun B. Dwaraka, Ryan Smith, Sara C. Zapico

**Affiliations:** 1 Department of Chemistry and Environmental Science, New Jersey Institute of Technology, 161 Warren St., Tiernan Hall 365, Newark, NJ 07102, USA; mc997@njit.edu (M.J.C.); mayaaskhassan@gmail.com (M.H.); 2 TruDiagnostic, Inc., 881 Corporate Dr, Lexington, KY 40503, USA; varun.dwaraka@trudiagnostic.com (V.B.D.); ryan@trudiagnostic.com (R.S.); 3 Anthropology Department and Laboratories of Analytical Biology, National Museum of Natural History, Smithsonian Institution, 10th and Constitution Ave., NW, Washington, DC 20560, USA

**Keywords:** smoking, vaping, buccal cells, exercise, DNA methylation

## Abstract

Background/Objectives: Lifestyle behaviors such as smoking, vaping, and physical activity can induce epigenetic modifications that influence health trajectories and may provide forensic value. DNA methylation signatures linked to these behaviors offer potential for behavioral inference, personalized health assessment, and improved investigative practices. This study aimed to characterize methylation patterns associated with nicotine exposure and exercise using buccal cell DNA profiling, and to evaluate the extent to which these patterns differentiate harmful and protective lifestyle habits. Methods: Buccal epithelial DNA was analyzed using the Illumina Infinium MethylationEPIC v2 BeadChip to assess genome-wide methylation. Participants were categorized by smoking status, vaping behavior, and exercise activity. Differentially methylated regions (DMRs) and CpG sites were identified through pairwise comparisons among smokers, vapers, non-smokers/non-vapers, athletes, and sedentary individuals. A threshold of *p* < 1 × 10^−4^ was applied for significant differentially methylated CpG sites. Results: Distinct epigenetic profiles were associated with smoking/vaping and physical activity. Five DMRs differentiated smokers from non-smokers/non vapers, while 11 DMRs distinguished vapers from the same reference group. Twenty-eight DMRs displayed divergent methylation patterns between smokers and vapers. Exercise also showed measurable epigenetic influence: control athletes exhibited 26 significantly differentially methylated CpG sites relative to non-athletes, and smoker athletes demonstrated 126 suggestive differential sites compared to sedentary smokers. Additionally, 63 sites differentiated smoker athletes from non-smoker/non-vaper non-athletes, indicating interactions between risk-associated and health-promoting behaviors. Conclusions: Buccal cell DNA methylation profiling effectively captured signatures associated with smoking, vaping, and physical activity. These findings underscore the potential of epigenetic markers for lifestyle assessment in both personalized medicine and forensic investigations.

## 1. Introduction

According to the latest World Health Organization (WHO) report, there are approximately 1.2 billion tobacco smokers older than 15 years old and 38 million adolescent smokers worldwide [1]. This tobacco epidemic leads to over 7 million deaths annually, as well as disability and long-term suffering from tobacco-related diseases [1,2]. Presenting as a harmless alternative to tobacco, Electronic Nicotine Delivery Systems (ENDS) or e-cigarettes (e-cigs) are increasing their popularity, particularly among young adults, with an estimated 35 million users globally [3,4]. These e-cigs use battery-operated non-combustible tobacco products, generating vapor through heating and aerosolizing nicotine, propylene glycol, and other products, which will be inhaled [3]. Due to the novelty of e-cigs, there are a few studies assessing their effects on health, preventing the elucidation of the long-term consequences of their use. Possibly, the most well-known disease directly associated with e-cigs is EVALI (E-cigarette or Vaping-Associated Lung Injury) [5]. It presents an acute pulmonary inflammatory process, producing alveolar collapse, and finally developing respiratory failure. Further studies indicated that the presence of vitamin E acetate and pre-existing conditions could be the trigger of the disease [6,7]. Apart from EVALI, researchers have reported the potential effects of e-cigs in the respiratory, cardiovascular, gastrointestinal, and nervous systems and oral cavity, some of them overlapping with tobacco smoking [4,8,9,10]. Still, more research is needed in this area to better understand their long-term impact.

One of the current useful biomarkers in tobacco smoking is one of the epigenetic modifications, DNA methylation (DNAm), the addition of a methyl group to a cytosine in cytosine–guanine sites located throughout the genome (CpGs) [11]. DNAm can reflect recent and longer tobacco smoke exposure, identifying methylated genes related to lung function, cancer, oxidative stress, and inflammation [12]. Some of these modifications revert after smoking cessation, while others remain [11]. There have been numerous studies assessing DNAm changes in tobacco smokers [11,12,13,14,15], some of them with forensic science application [16,17,18,19,20,21,22].

One of the main objectives of forensic science is the identification of the perpetrator from a body fluid left at the crime scene [23]. However, when there is not a reference sample to compare with a suspect or on the database, it would be useful to obtain as much information as possible from the body fluid: eye, hair, and skin color [24], among others, as well as age. There is extensive research related to the application of DNA methylation for age estimation in forensic sciences, mainly with criminalistic purposes, from blood, semen, and saliva left from the perpetrator at the crime scene [25]. These studies and previous knowledge pointed out how lifestyle could affect DNA methylation, triggering further research assessing the possibility of determining the smoking status of a person based on blood and saliva samples through DNA methylation. Many of these studies used the Illumina Infinium Methylation microarrays [17,18,19,21,22], while others applied massive parallel sequencing [20] or pyrosequencing, evaluating only a few genes and methylation sites [16]. Many of them pointed out that the methylation of *AHRR* (Aryl Hydrocarbon Receptor Repressor) is the main predictor for smokers in both types of samples, in combination with other genes, like the *F2RL3* (coagulation factor II (thrombin) receptor-like 3), though, depending on the model. Despite this extensive research on smoking, from a forensic point of view, there are no current studies focusing on the epigenetic signatures in vaping. Though from the clinical perspective, studies are emerging evaluating DNA methylation signatures between smoking and vaping, finding commonalities and differences, which could help to determine the long-term effects of e-cigs [11,12,14,15,26]. This is the first gap in vaping research: it is crucial to develop further studies in this field to elucidate these effects, with smoking findings as a reference.

Another important lifestyle factor is exercise. Several studies demonstrated the benefits of exercise at the epigenetic level [27], and particularly on DNA methylation [28], in health maintenance and disease prevention [29,30], and reducing epigenetic aging in combination with other interventions [31,32]. However, there are no studies assessing the impact of exercise on smokers and vapers from the clinical or forensic perspective. The only ones developed in clinic evaluated skeletal muscle and cardiorespiratory functions [9,10,33,34], or they are sociological studies to uncover a potential association among e-cig use and physical activity [35,36,37,38]. None of these works simultaneously analyzed smoking, vaping, and the impact of exercise at the epigenetic level. This is the second gap in smoking/vaping research. Due to the flexible nature of epigenetics and potential benefits of exercise, it is possible that the latter potentially overcomes some of the effects of smoking/vaping. As a result, it is imperative to carry out studies in this area.

Considering the aforementioned gaps (the few studies evaluated methylation changes and the lack of research on the effect of exercise in smokers and vapers), the objective of this study was to evaluate DNA methylation signatures of smoking and vaping in a young adult population (18–31 years old) and assess the impact of exercise on these patterns.

## 2. Material and Methods

### 2.1. Study Participants

One hundred eighty participants aged 18 to 31 years were recruited from the New Jersey Institute of Technology student population (Newark, NJ, USA) in 2025. The sample was restricted to this age range to target the young adult population, because the study was conducted within a university setting, enabling the inclusion of individuals with diverse lifestyle habits relevant to the study, including athletes, smokers, and vapers. This age group is also commonly used in biomedical and forensic research due to the lower prevalence of age-related conditions. Lifestyle information was collected via a structured questionnaire that covered active smoking or vaping and the duration, alcohol and drug consumption and frequency, and physical activity, including the type of exercise (individual or team sports, simple workouts) (Supplementary Table S1). Smokers and vapers were included if they used these products frequently for the past 6 months.

Fifteen participants (8.4% of the cohort) reported both smoking and vaping (“dual users”). For classification purposes, dual users were assigned to the “Smoker” group to prioritize the more harmful combustible tobacco exposure, as cigarette smoke contains >7000 chemicals including potent carcinogens whereas e-cigarettes contain fewer toxicants [3]. Thus, the “Vaper” group (n = 12) represents exclusive e-cigarette users with no concurrent cigarette smoking, while the “Smoker” group (n = 38) includes both exclusive smokers (n = 23) and dual smoker/vapers (n = 15). Duration data were available for 35/38 smokers (92%, mean of 3.2 ± 2.1 years, range from 1 month to 9 years) and 11/12 vapers (92%, mean of 2.1 ± 2.3 years). Quantitative intensity data (cigarettes per day, vaping frequency/pods per week, pack-years) were not systematically collected, limiting our ability to perform dose–response analyses. This is acknowledged as a study limitation (see Discussion Section).

Exclusion criteria included dependence on alcohol or drugs (other than nicotine), significant current or past illness or conditions, current pregnancy or breastfeeding, and having a related individual in the sample. The study received approval from the New Jersey Institute of Technology Institutional Review Board (IRB protocol no. 2110013076R003), and all participants provided written informed consent.

Based on these data, participants were categorized into three groups: (i) a control group (n = 129), defined as individuals who never smoked or vaped regularly, further subdivided into 81 sports-active and 48 non-active subjects; (ii) an active smokers group (n = 38), comprising 29 sports-active and 9 non-active individuals; and (iii) an active vapers group (n = 12), consisting of 9 sports-active and 3 non-active subjects (Table 1).

### 2.2. Sample Collection

Buccal epithelial cell samples were collected from the inner right and left cheeks with swabs (McKesson, Richmond, VA, USA), through circular movements for 20 s. Two samples (one per cheek) were obtained per participant.

### 2.3. DNA Extraction and Quantification

Genomic DNA was extracted from one of the samples using the DNeasy^®^ Blood and Tissue Kit (Qiagen, Hilden, Germany), a silica-membrane column–based system, following the manufacturer’s instructions, and eluted in a final volume of 35 µL of AE buffer. Negative controls (extraction blanks containing only elution buffer) were processed alongside samples at a ratio of 1:20 to monitor potential contamination. All negative controls yielded DNA concentrations below detection limit (<1 ng/µL), confirming no cross-contamination. DNA concentrations obtained from the samples ranged between 6.12 and 60 ng/µL. To meet the minimum DNA input requirement of 200 ng for EPIC array processing, a standardized volume of 33 µL per sample was used. DNA quantification was performed by fluorometry using the Qubit™ Fluorometer 1.0 (Thermo Fisher Scientific, Waltham, MA, USA) with the Qubit™ dsDNA High Sensitivity Assay (Life Technologies, Carlsbad, CA, USA). Positive controls (known sample concentrations) and negative controls were included in these steps.

### 2.4. DNA Methylation Profiling

DNA samples were sent to TruDiagnostics (Lexington, KY, USA). Bisulfite conversion was performed using the EZ DNA Methylation™ Kit (Zymo Research, Irvine, CA, USA), following the manufacturer’s standard protocol. Briefly, 500 ng of genomic DNA per sample was treated with sodium bisulfite to convert unmethylated cytosines to uracils while leaving methylated cytosines unchanged. All samples passed Illumina’s recommended quality metrics (detection *p*-value < 0.01 for >95% of probes). Genome-wide methylation status of over 930,000 CpGs sites was measured with the Infinium MethylationEPIC BeadChip v2 (Illumina, San Diego, CA, USA) of 179 DNA samples, since 1 sample from the control non-sports group did not meet quality control and was excluded. Data preprocessing and normalization were carried out in R (v4.4.0) using the SeSAMe package (v1.29.2) with the ssNoob method.

### 2.5. Statistical Analysis

A cross-sectional epigenome-wide association study (EWAS) was conducted using linear models with empirical Bayes moderation in the limma package (v3.67.0). Statistical models were adjusted for sports activity, decimal chronological age, binary sex, binary alcohol consumption, and beadchip batch effects.

Two complementary analytical approaches were employed: (1) individual CpG site analysis using limma, and (2) regional analysis using DMRcate to identify differentially methylated regions (DMRs) consisting of 3 or more consecutive CpGs showing consistent directional changes. These approaches address different biological questions: individual CpG analysis detects site-specific methylation changes, while DMR analysis identifies broader genomic regions with coordinated methylation alterations, which may have greater functional relevance.

For individual CpG site analysis, genome-wide significance was assessed using the Benjamini–Hochberg False Discovery Rate (FDR), with adjusted *p* values < 0.05 considered definitive (FDR-significant). An additional discovery threshold of *p* < 1 × 10^−4^ was used to identify suggestive associations in comparisons with limited statistical power (particularly sports-stratified subgroup analyses with small sample sizes, n < 20 in some groups), where FDR-corrected significance was not achieved. These discovery-level hits (*p* < 1 × 10^−4^, uncorrected) are considered exploratory and hypothesis-generating, requiring independent replication. Throughout the Results Section, we explicitly distinguish between FDR-significant findings (definitive associations) and discovery-threshold findings (suggestive associations). Differentially methylated regions (DMRs) were identified using the DMRcate package in R (v4.4.0) [39], which applies Gaussian kernel-smoothed estimates to combine statistical evidence from spatially correlated neighboring CpG sites. DMRs were called using default parameters: lambda (bandwidth) = 1000 nucleotides, scaling factor C = 2. Regions were required to contain a minimum of 3 CpGs with consistent directional methylation changes (all hypermethylated or all hypomethylated). Statistical significance was assessed using Stouffer’s Z-score method to combine *p*-values across CpGs within each region, with Stouffer FDR < 0.05 considered significant. DMR analysis was performed only for the three primary comparisons with adequate statistical power: smokers vs. non-smokers/non-vapers (n = 38 vs. 129), vapers vs. non-smokers/non-vapers (n = 12 vs. 129), and smokers vs. vapers (n = 38 vs. 12). Sports-stratified comparisons utilized individual CpG analysis due to smaller subgroup sample sizes (n = 3–9 in some groups).

All CpGs and DMRs were annotated with genomic context using the IlluminaHumanMethylationEPICanno.ilm10b4.hg19 Bioconductor package (reference genome: hg19/GRCh37) (v0.6.0). Annotations include: (1) Gene feature: Promoter (TSS-1500 to TSS + 500), 5′UTR, 1st Exon, Gene Body, 3′UTR, or Intergenic (>1.5 kb from any annotated gene); and (2) CpG island context: Island (within CpG island), N_Shore/S_Shore (within 2 kb upstream/downstream of island), N_Shelf/S_Shelf (2–4 kb from island), or OpenSea (>4 kb from nearest island). Genomic location influences functional interpretation: promoter/5′UTR methylation typically represses transcription, gene body methylation may enhance expression, and intergenic methylation may affect distal regulatory elements (enhancers, silencers) controlling genes located hundreds of kilobases to megabases away through chromatin looping. Therefore, gene names serve primarily as genomic landmarks rather than definitive predictions of affected gene expression.

**Figure 1:**
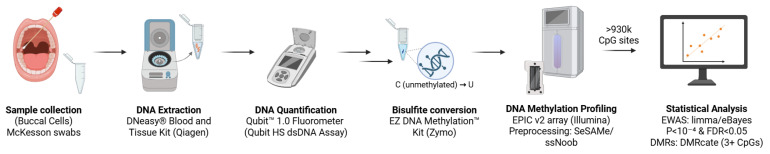
Overview of the study workflow for DNA methylation analysis. DNA methylation profiling was performed at TruDiagnostics (Lexington, KY, USA). Figure created with BioRender.com.

A summary of the methods carried out in this study is depicted in Figure 1.

## 3. Results

Based on the size differences among study groups, it is important to note that the findings of this study should be considered exploratory.

### 3.1. Epigenetic Wide-Association Studies (EWAS) Among Non-Smokers/Non-Vapers, Smokers, and Vapers

DMRs were assessed for these comparisons, focusing on statistical significance based on Stouffer FDR, mean difference, and number of CpGs in the region.

#### 3.1.1. Smokers vs. Non-Smokers/Non-Vapers

The genomic inflation coefficient for this comparison was λ = 0.86. We identified five differentially methylated regions (DMRs) associated with smoking status (FDR < 0.05). The most significant DMR overlap to a gene was located in the *CPXM2* gene (chr10:123922970-123923099), showing 4.53% lower methylation in smokers compared to non-smokers/non-vapers controls (FDR = 8.2 × 10^−5^, 5 CpGs). Additional DMRs were identified in *TYMP* (chr22:50527953–50528641, promoter region within CpG island, 2.4% hypomethylated in smokers, Stouffer FDR = 0.014) and *SMAD9* (gene body, CpG shore, hypomethylated in smokers, Stouffer FDR = 0.032). All five DMRs showed consistent directional changes across constituent CpGs (Supplementary Table S2). Note: Gene names denote genomic location only; functional effects may involve distant genes regulated by these differentially methylated regions, particularly for CpGs in intergenic regions that may affect enhancers or silencers controlling genes located hundreds of kilobases away.

#### 3.1.2. Vapers vs. Non-Smokers/Non-Vapers

The genomic inflation coefficient for this comparison was λ = 0.79. We identified 11 DMRs associated with vaping status (FDR < 0.05). The most significant DMR was located in the *AP3B1* gene (chr5:78281103-78282099), within the promoter region (TSS-200) in a CpG island, showing 9.20% higher methylation in vapers compared to non-smokers/non-vapers (Stouffer *p* = 6.74 × 10^−11^, FDR = 0.001, 10 CpGs). Promoter hypermethylation is typically associated with transcriptional silencing. Additional DMRs were identified in *OR3A2* (chr17:3289363–3290164; 8 CpGs; +5.4% methylation; HMFDR = 0.00304) and *FAM50B* (chr6:3848243–3849818; 27 CpGs; +4.9% methylation; Stouffer FDR = 1.58 × 10^−35^), the latter overlapping *AL391422.3* and *AL391422.2* (Supplementary Table S2).

#### 3.1.3. Smokers vs. Vapers

The genomic inflation coefficient for this comparison was λ = 0.80. We identified 28 DMRs between smokers and vapers (FDR < 0.05). The most significant DMR was located in the *GLCE* gene (chr15:69222400-69223774), spanning 1.4 kb in the gene body within an OpenSea region (>4 kb from CpG islands), showing 6.90% lower methylation in smokers compared to vapers (Stouffer *p* = 1.44 × 10^−14^, FDR = 0.008, 9 CpGs). Notable DMRs were also observed in *FAM50B* and *KLHDC10*, both showing lower methylation in smokers compared to vapers (Supplementary Table S2).

**Figure 2:**
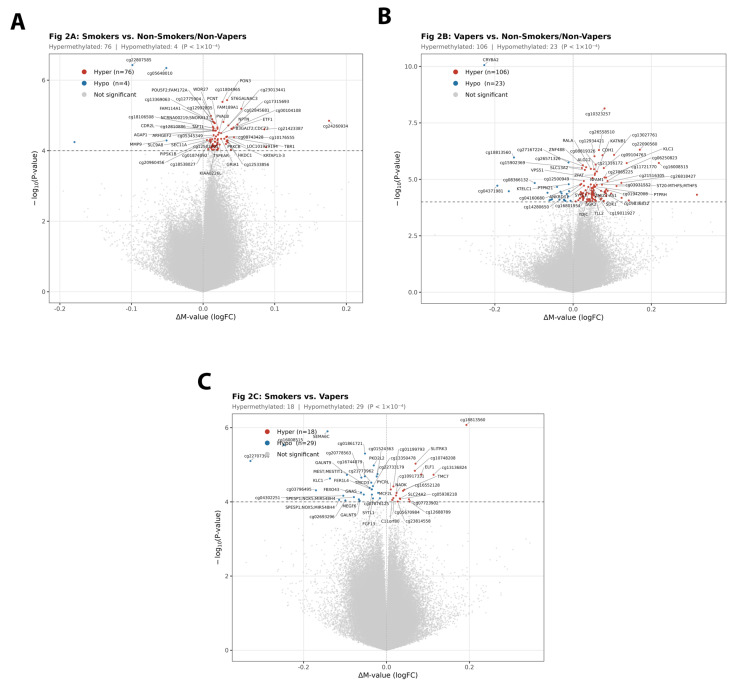
Volcano plots depicting differentially methylated CpG sites. (**A**) Smokers (n = 38) vs. non-smokers/non-vapers (n = 129). (**B**) Vapers (n = 12) vs. non-smokers/non-vapers (n = 129). (**C**) Smokers (n = 38) vs. vapers (n = 12). Each point represents one CpG site. *X*-axis: logFC (log_2_ fold change in M-values); positive values indicate hypermethylation in the first group, and negative values indicate hypomethylation. *Y*-axis: -log_10_(*p*-value). **Red points**: Hypermethylated CpGs (logFC > 0, *p* < 1 × 10^−4^). **Blue points**: Hypomethylated CpGs (logFC < 0, *p* < 1 × 10^−4^). **Gray points**: Non-significant (*p* ≥ 1 × 10^−4^). Top 10 significant CpGs labeled with gene symbol (if annotated) or probe ID (if intergenic, denoted with “(intergenic)” notation). Horizontal dashed line: *p* = 1 × 10^−4^ discovery threshold. Note: Gene names indicate nearest gene for genomic reference only; intergenic CpGs may affect distal regulatory elements.

**Figure 3:**
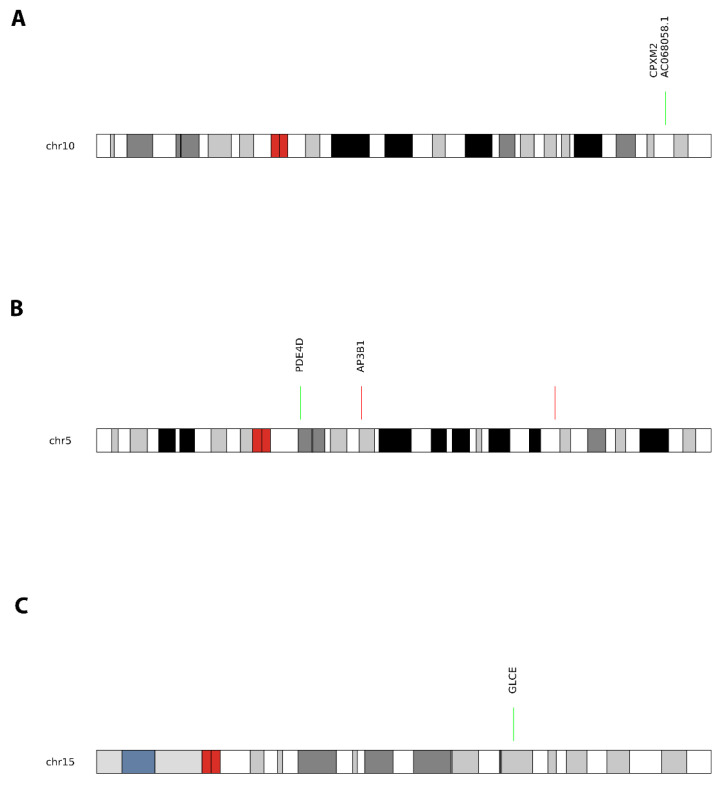
Top differentially methylated regions (DMRs). (**A**) Smokers vs. non-smokers/non-vapers: *CPXM2* (chr10:123922970-123923099, gene body, CpG shore, 4 CpGs). (**B**) Vapers vs. non-smokers/non-vapers: *AP3B1* (chr5:78281103-78282099, promoter region, CpG island, 10 CpGs). (**C**) Smokers vs. vapers: *GLCE* (chr15:69222400-69223774, gene body, OpenSea, 9 CpGs). Each panel shows: chromosome ideogram (top track); methylation levels (β-values, 0–1 scale) by sample group with mean ± SE; gene models (UCSC RefGene); CpG island annotations. Statistical significance (Stouffer FDR < 0.05) indicated. DMRs: regions of 3+ consecutive CpGs with coordinated methylation changes. Genomic coordinates: hg19 genome build.

Figure 2 depicts volcano plots of these comparisons. Figure 3 shows the top DMRs overlapping with gene regions between smokers and non-smokers/non-vapers; vapers and non-smokers/non-vapers; smokers and vapers. Table 2 summarizes these findings.

#### 3.1.4. Investigation of *AHRR cg05575921*

We specifically examined *AHRR cg05575921* (chr5:373378), the most widely replicated smoking biomarker in adult cohorts. This probe passed quality control and was included in all analyses. Contrary to expectations from studies of older, long-term smokers, *AHRR* showed no significant hypomethylation in smokers versus non-smokers/non-vapers in our young adult cohort (mean β-value: smokers = 0.754 ± 0.070, non-smokers = 0.767 ± 0.062; difference = −1.3%, *p* = 0.353).

To investigate whether physical activity modified the smoking effect on *AHRR*, we tested a smoking × sports interaction model (M-value ~ Smoking * Sports + Age + Sex). The interaction term was non-significant (*p* = 0.442), though we observed a pattern suggesting potential attenuation: smokers who were sedentary showed −2.9% lower AHRR methylation relative to healthy controls, whereas smokers who exercised showed only −0.7% lower methylation (+2.2% attenuation, underpowered to detect significance).

The absence of significant *AHRR* hypomethylation in our cohort likely reflects three factors: (1) Young age and short smoking duration: Our participants (18–31 years, mean smoking duration of 3.2 ± 2.1 years) have substantially less cumulative exposure than cohorts where *AHRR* robustly differentiates smokers (typically 10+ years duration, 20+ pack-years). *AHRR* methylation changes are cumulative and require extended exposure [40]. (2) High sports participation: A total of 76% of our smokers (29/38) were athletes. Pospiech et al. [22] demonstrated that AHRR methylation is responsive to physical activity, which may attenuate smoking-induced hypomethylation. (3) Tissue and developmental factors: Buccal cells from young adults may exhibit age-dependent methylation trajectories distinct from blood or older adult tissues.

Importantly, the non-significance of *AHRR* does not invalidate our findings. Rather, our significant DMRs (*CPXM2*, *TYMP*, *SMAD9*) represent early epigenetic responses to smoking detectable within 1–3 years of exposure initiation, distinct from cumulative chronic-smoking biomarkers like *AHRR* that emerge with longer duration. See Supplementary Figure S1 and Supplementary Table S4 for detailed *AHRR* analysis.

### 3.2. Epigenetic Wide-Association Studies (EWAS) Considering Physical Activity

The following sports-stratified comparisons utilized individual CpG analysis at discovery threshold *p* < 1 × 10^−4^ (uncorrected). Due to small sample sizes in several subgroups (e.g., Smokers_No_Sports n = 9, Vapers_No_Sports n = 3), DMR analysis was not performed. Results are considered exploratory and hypothesis-generating, intended to guide future studies with larger sample sizes. Only one comparison (Vaper_Sports vs. Healthy_No_Sports) yielded CpGs reaching FDR < 0.05 significance (n = 4 CpGs), likely reflecting the confounded nature of this comparison (mixing both substance use and activity level differences). Genomic inflation coefficients (λ) are reported for each comparison to assess test calibration; values <0.8 indicate overly conservative tests (often due to sample size imbalance and covariate adjustment), while values > 1.1 suggest adequate power.

#### 3.2.1. Non-Smokers/Non-Vapers No Sports vs. Sports

**Figure 4:**
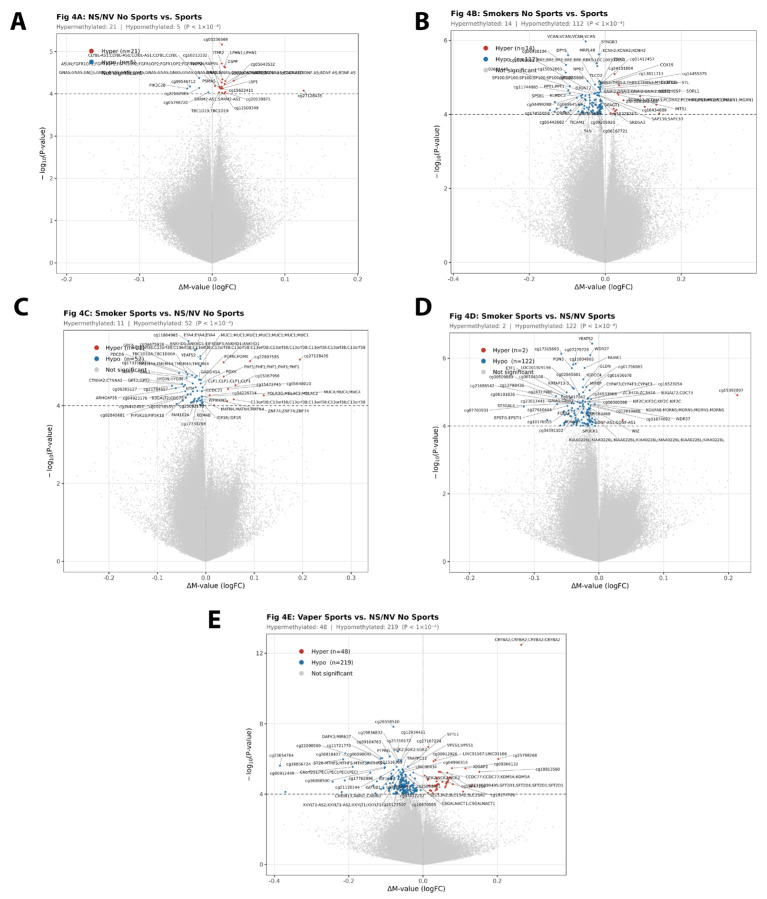
Volcano plots for sports-stratified comparisons (discovery threshold *p* < 1 × 10^−4^, exploratory). (**A**) Non-smokers/Non-vapers: No Sports (n = 48) vs. Sports (n = 81). (**B**) Smokers: No Sports (n = 9) vs. Sports (n = 29). (**C**) Smoker_Sports (n = 29) vs. Non-smokers/Non-vapers_No_Sports (n = 48). (**D**) Smoker_Sports (n = 29) vs. Non-smokers/Non-vapers Sports (n = 81). (**E**) Vaper_Sports (n = 9) vs. Non-smokers/Non-vapers_No_Sports (n = 48). Format as Figure 2: logFC = log_2_ fold change in M-values; positive = hypermethylation in first group. **Red**: hypermethylated CpGs (*p* < 1 × 10^−4^). **Blue**: hypomethylated CpGs (*p* < 1 × 10^−4^). **Gray**: non-significant. λ = genomic inflation coefficient (shown on each plot); values < 0.8 indicate conservative tests due to small sample sizes and covariate adjustment. **IMPORTANT**: These comparisons report discovery-level hits (*p* < 1 × 10^−4^, uncorrected); only Panel E yielded CpGs reaching FDR < 0.05 (n = 4 CpGs). Results are exploratory and require replication.

Among non-smokers/non-vapers, 81 practice any kind of sport/exercise, and 48 do not practice any sports/exercise. Based on the number of subjects and genomic inflation coefficient (λ = 1.003), this comparison had sufficient statistical power to detect suggestive association. In total, 26 CpGs met the discovery threshold (*p* < 1 × 10^−4^, uncorrected; none reached FDR < 0.05), though only one presented a suggestive and large effect, cg27128435, with more than 10% hypermethylation difference. *PIK3C2B* and cg09546712 presented 2–5% hypomethylation difference, as well as cg05643532, hypermethylated. The rest of the hits showed small effects sizes less than 2% (Supplementary Table S3). Figure 4A depicts the volcano plot of these data, highlighting suggestive significant hypo- and hypermethylated CpGs.

#### 3.2.2. Smokers No Sports vs. Smokers Sports

Among smokers, 29 practice any kind of sport/exercise and 9 do not practice any sports/exercise. Despite the genomic inflation coefficient (λ = 1.092), this comparison has limited statistical power due to the small sample size in the no-sports group (n = 9). Results should be interpreted cautiously and considered exploratory. In total, 126 discovery hits (*p* < 0.0001) were found, predominantly hypomethylated (112) with more than 10% methylation difference in cg26938104 (hypomethylated), cg15052665 (hypomethylated), *CYYR1* (hypomethylated), cg25907935 (hypermethylated), *DGKB* (cg18714845, hypermethylated), cg16921836 (hypomethylated), *TRIM10* (cg11079936, hypomethylated), cg11704064 (hypomethylated), *YWHAG* (cg00347643, hypomethylated), *GRM1* (cg23376330, hypomethylated), cg13391142 (hypomethylated), cg18660106 (hypermethylated), cg27478961 (hypomethylated). *VCAN (cg01061880)*, *DPYS (cg01514867)*, *SP100 (cg16987437)*, *PPT1 (cg18212197)*, *SPSB1 (cg19820919)*, *KLHDC2 (cg01941274)*, *OR6K6 (cg01300866)*, cg04499288, cg06864533, cg17455058, cg01442682, *TICAM1* (cg07550579), *FSD1* (ch.19.233112R), *GPATCH2L* (cg25474288), cg00591228, *LMO7(cg18027592)*, cg22855900, *PCDHGA4 (cg02022808)*, *FAM190A (cg14834881)*, *ARHGAP26 (cg00618275)*, *TCF7L2 (cg19084061)*, and cg16402816 were hypomethylated with a difference in the range of 5–10%, while *PCDHA3 (cg21768610)* was found hypermethylated. The rest of the hits showed less than 5% methylation difference (Supplementary Table S3). Figure 4B depicts the volcano plot of these data, highlighting suggestive significant hypo- and hypermethylated CpGs.

#### 3.2.3. Smokers Sports vs. Non-Smokers/Non-Vapers No Sports

The statistical comparison of these groups was limited by the number of samples (29 smokers who practice sports vs. 48 non-smokers/non-vapers who do not practice sports) and a deflated genomic inflation coefficient (λ = 0.721), suggesting the model may be overly conservative due to the sample size imbalance and covariate adjustment. In total, 63 discovery hits (*p* < 0.0001) were found, predominantly hypomethylated (52), although the largest changes (>10%) were hypermethylated at cg27128435 and *MUC4 (cg08521684)*, both hypermethylated. In contrast, *GRIK4 (cg22483170)*, *ARHGAP35 (cg23719905)*, cg06385127, and cg17315693 were hypomethylated with a difference in the range of 5–10%, while cg22807585, cg15167956, and *C13orf38* (cg11678027) were found hypermethylated. The rest of the hits showed less than 5% methylation difference (Supplementary Table S3). Figure 4C depicts the volcano plot of these data, highlighting suggestive significant hypo- and hypermethylated CpGs.

#### 3.2.4. Smokers Sports vs. Non-Smokers/Non-Vapers Sports

A total of 81 non-smokers/non-vapers and 29 smokers practice sports regularly. Based on the number of subjects and genomic inflation coefficient (λ = 1.122), this comparison has excellent power to carry out this analysis. This comparison isolates the effect of smoking within the sports-active population. Both groups exercise regularly, so differences reflect smoking effects independent of physical activity level. In total, 124 discovery hits (*p* < 0.0001) were found, predominantly hypomethylated (122) with more than 10% methylation difference in cg15392097 (hypermethylated) and cg07703931 (hypomethylated). A total of 5–10% hypomethylation differences were found in *ASTN2 (cg08153316)*, cg00509890, cg13499721, cg21191371, *CHN2 (cg17822452)*, *B2M (cg27537252)*, cg08743428, *NPTN (cg11138503)*, *ATP8A1 (cg13212407)*, cg27610444, cg13798436, cg00509889, and cg17315693. The rest of the hits showed less than 5% methylation difference (Supplementary Table S3). Figure 4D depicts the volcano plot of these data, highlighting suggestive significant hypo- and hypermethylated CpGs.

#### 3.2.5. Vapers Sports vs. Non-Smokers/Non-Vapers No Sports

The statistical comparison of these groups was limited by the number of samples (9 vapers practicing sports vs. 48 non-smokers/non-vapers not practicing sports) and a deflated genomic inflation coefficient (lambda = 0.791), indicating a conservative test likely driven by the small vaper group size and covariate adjustment. In total, 267 discovery hits (*p* < 0.0001) were found, with more than 10% hypermethylation difference in *CRYBA2* (cg02805994), cg25798268, cg08366132, and cg18813560. In contrast, cg16088500, *C6orf201*, cg00912449, cg23654764, cg06098042, ST20-MTHFS (cg12376289), cg26818427, cg18856724, cg11721770, *DAPK3 (cg27028514)*, and cg22090560 were found with more than 10% hypomethylation difference. Hypomethylation differences in the range of 5–10% were discovered in cg04990314, *LINC00434* (cg27492296), *VPS51 (cg19901918)*, *KATNB1 (cg00478908)*, cg21120144, *TRAPPC12 (cg00086186)*, cg21516305, *SGK2 (cg23820983)*, *PTPRG (cg03844714)*, cg12934421, cg19836832, cg09104763, cg21316172, and cg26558510, while only *IQGAP2 (cg11051031)* presented hypermethylation differences in the range of 5–10% (Supplementary Table S3). More discoveries were found with a 5–10% methylation difference, but they are not significant. The rest of hits showed less than 5% methylation difference. Figure 4E depicts the volcano plot of these data, highlighting suggestive significant hypo- and hypermethylated CpGs.

## 4. Discussion

DNA methylation is emerging as a biomarker of environmental and lifestyle factors [30]. Among them, there is a body of evidence of the impact of tobacco on DNA methylation patterns, with these changes lasting even after cessation and connected with the development and prognosis of smoking-associated diseases [13]. In contrast to tobacco smoking, little is known about the long-term effects of e-cigarettes [4,8,41]. They are sold as a safer alternative to tobacco, gaining popularity mostly among young people and smokers who would like to quit [42,43,44]. Currently, there are a few studies assessing DNA methylation changes in vapers, also compared to tobacco smoking, which could help to understand their long-term effects and be a biomarker of e-cigs exposure [11,12,14,26].

Our study aimed to contribute to these previous works, evaluating DNA methylation patterns of smoking and vaping in young adults, since this is the target population for e-cigs initiation. Regarding the absence of *AHRR* (aryl hydrocarbon receptor repressor, cg05575921) as a significant differentially methylated locus, we conducted a thorough investigation to address this unexpected finding (see Results Section 3.1.4 and Supplementary Table S4/Figure S1). *AHRR cg05575921* passed quality control and was present in our dataset but showed no significant hypomethylation in smokers versus non-smokers/non-vapers (mean difference of −1.3%, *p* = 0.353).

We propose three non-mutually-exclusive explanations: (1) Young age and short smoking duration: Our cohort (aged 18–31 years, median smoking duration of 3.2 years) is substantially younger than cohorts where *AHRR* robustly differentiates smokers (typically aged 40–70 years, 20+ pack-years). *AHRR* methylation changes are cumulative and increase with smoking duration at approximately −0.3% per pack-year [40]. Our participants’ brief exposure (estimated 1–5 pack-years) may be insufficient to produce detectable *AHRR* changes. (2) Physical activity attenuation: Critically, 76% of our smokers (29/38) were athletes. Pospiech et al. [22] demonstrated that *AHRR* methylation is responsive to physical activity (+2.1% in active individuals), suggesting exercise may partially offset smoking-induced hypomethylation. Our smoking × sports interaction analysis revealed a non-significant trend (*p* = 0.44): sedentary smokers showed −2.9% lower *AHRR* methylation versus healthy controls, while athletic smokers showed only −0.7% lower methylation (+2.2% attenuation). (3) Tissue-specific developmental patterns: Buccal cells from young adults exhibit age-dependent methylation trajectories distinct from blood or older adult tissues.

Importantly, the absence of AHRR hypomethylation does not invalidate our findings. *AHRR* is a late-stage, cumulative biomarker requiring years of exposure. Our significant DMRs (*CPXM2*, *TYMP*, *SMAD9*) represent early epigenetic responses to smoking detectable within 1–3 years of exposure initiation, a previously understudied phase. This suggests a temporal progression: early-stage biomarkers (captured in our study) precede late-stage cumulative biomarkers like *AHRR.* Our findings provide novel insights into the initial epigenetic perturbations following smoking initiation in young adults.

Despite this result, in the comparison of smokers vs. non-smokers/non-vapers, other DMRs located in genes were found to be significantly hypomethylated in smokers, though this does not mean that the listed gene is necessarily affected by the DNA methylation change. *CPXM2* (Carboxypeptidase X, M14 Family Member 2) is involved in proteolysis, and previous studies relate its overexpression with cardiac hypertrophy and failure [45] and unfavorable prognosis in different cancers [46,47]. *SMAD9* (SMAD Family Member 9) transduces signals from TGF-beta family members. Previous works correlate its genetic variations with unfavorable overall survival in non-small cell lung cancer [48]. *TYMP* (Thymidine Phosphorylase) is also involved in the prognosis of various cancers [49] and metabolism of chemotherapeutic drugs [50], associating certain polymorphisms with the risk of chemotherapy-induced toxicity [51]. Overall, significant DMRs found between smokers and non-smokers/non-vapers are related to proteolysis, cell growth and migration, immune regulation, and drug metabolism.

Notably, the genomic context of these DMRs provides insights into potential mechanisms. The *CPXM2* DMR occurs in a gene body CpG shore region, where hypomethylation typically correlates with reduced gene expression [52], suggesting potential downregulation in smokers. In contrast, the *TYMP* DMR is located in a promoter CpG island and shows hypomethylation in smokers, consistent with transcriptional activation. Among our 44 total DMRs across all three primary comparisons, 43.2% (19/44) occur in intergenic regions where methylation changes are more likely to affect regulatory elements controlling distant genes rather than the nearest annotated gene [53].

Comparison between vapers and non-smokers/non-vapers found mostly hypermethylated DMRs in vapers. The strongest one was in *AP3B1* (Adaptor Related Protein Complex 3 Subunit Beta 1), which plays a role in organelle biogenesis associated with melanosomes, platelet dense granules, and lysosomes. Its genetic variants are associated with Hermansky–Pudlak syndrome type 2, producing, among others, immunodeficiency, directly related to these variants [54,55]. *FAM50B* (Family with Sequence Similarity 50 Member B) is imprinted and paternally expressed in many tissues [56]. Previous studies demonstrated its hypermethylation after air pollution exposure [57], and its involvement in cancer prognosis [58,59,60]. *OR3A2* (Olfactory Receptor Family 3 Subfamily A Member 2) interacts with odorant molecules in the nose to initiate a neuronal response that triggers the perception of a smell. The olfactory receptor proteins are members of a large family of G-protein-coupled receptors (GPCRs) arising from single coding-exon genes [61]. No previous studies related this receptor to smoking/vaping or related diseases, though it is reasonable to expect that olfactory receptors would be affected by e-cigarette exposure.

Comparison between smokers and vapers also revealed three main DMRs, in this case hypomethylated in smokers. In contrast to the previous comparison, *FAM50B* was found to be hypomethylated in smokers. The highest significant hypomethylation was found in *GLCE* (d-glucuronyl C5-epimerase), which enables calcium ion binding activity, and its expression has a prognostic value in different tumors [62,63,64]. *KLHDC10* (Kelch Domain Containing 10) enables ubiquitin-like ligase-substrate adaptor activity. Some studies pointed out to be a regulator of oxidative stress-induced apoptosis and inflammation [65,66], and its methylation was identified as a potential colorectal cancer risk biomarker [67]. A recent study in buccal cells from patients treated with glucocorticoids after dental surgery found hypomethylation of this gene [68]. Thus, significant DMRs between smokers and vapers are mostly associated with inflammatory and oxidative stress pathways.

These findings increase current knowledge of biomarker discovery for smoking and vaping status in both clinical [11,12,14,26] and forensic settings [17,18,19,20].

The second part of this study was to assess the potential impact of exercise on these groups. Previous works described DNA methylation adaptive changes in response to exercise [28,30], improving health [29], even in older adults, in combination with other therapies [31]. The first comparison was between non-smokers/non-vapers no sports vs. non-smokers/non-vapers sports, finding, among others, hypomethylated *PIK3C2B* (Phosphatidylinositol-4-Phosphate 3-Kinase Catalytic Subunit Type 2 Beta), involved in different processes of cell proliferation, oncogenic transformation, cell survival, cell migration, and intracellular protein trafficking. It has been found hypomethylated in certain cancers [69,70]. Several discovery hits were found with the comparison of smokers no-sports vs. smokers sports. Among them, it is worth to mention *YWHAG* (Tyrosine 3-Monooxygenase/Tryptophan 5-Monooxygenase Activation Protein Gamma), which previous studies related to smoking initiation [71]; *PPT1* (Palmitoyl-Protein Thioesterase 1), also associated with susceptibility for cigarette smoke in chronic obstructive pulmonary disease (COPD) [72]; and Tcf7L2 (transcription factor 7-like 2), related to cigarette smoking addiction and diabetes [73,74]. In the comparison of smokers sports vs. non-smokers/non-vapers no sports, the most significant discovery is *MUC4* (Mucin 4) hypermethylation in smokers sports, as in contrast, previous studies demonstrated an upregulation of MUC4 in smokers and respiratory diseases associated with smoking [75,76,77]. In the comparison of smokers sports vs. non-smokers/non-vapers sports, *B2M* (Beta-2-Microglobulin) and *ATP8* (ATPase Phospholipid Transporting 8A1) have been previously described as a potential marker of smoking status in different lung cell types [78]. *ASTN2* (Astrotactin 2), involved in neural migration, has been implicated in the regulation of emotional and cognitive functions [79]. In the comparison between vapers sports vs. non-smokers/non-vapers no sports, possibly the hypermethylation of *CRYBA2* (Crystallin Beta A2) was the most significant finding, as this gene has been related to the development of cataracts [80]. Moreover, *DAPK3* (Death-Associated Protein Kinase 3), hypomethylated, is a tumor-suppressor gene that regulates cell death [81,82]. These results point to a certain genetic reprogramming because of practicing sports; as some of these findings had low statistical power, they should be considered carefully.

Several potential confounders warrant consideration. First, buccal samples contain mixed cell populations (epithelial cells, immune cells) whose proportions may vary between groups; our models adjusted for age and sex as proxies for cell composition. Second, we lacked quantitative smoking/vaping intensity data, as discussed above. Third, batch effects were controlled by including beadchip as a covariate in all models. Fourth, DNA methylation is highly tissue-specific, and buccal findings may not generalize to blood or other tissues [83]. Despite these limitations, the internal consistency of our findings, biological plausibility of implicated pathways, dose–response patterns, and replication of known biology support the validity of our primary associations.

As noted above, this study is exploratory and based on buccal cell methylation profiles; therefore, potential tissue-specific effects must be considered. Numerous chromosomal loci—known as tissue-specific differentially methylated regions (tDMRs)—exhibit characteristic methylation signatures that vary across tissues and cell types [84,85,86,87]. These regions have been correlated with transcriptional activity, suggesting a functional role in gene regulation. However, the exact mechanisms by which tDMR methylation influences transcription remain only partially understood. Despite these uncertainties, substantial evidence indicates that hypermethylation within tDMRs is frequently associated with reduced gene expression or gene silencing [88]. Consequently, the findings reported in this paper should be interpreted with caution, as genes appearing differentially affected in buccal cells may not show comparable methylation changes in other tissues or biofluids, such as blood or lung tissue.

This study represents an initial step toward identifying biomarkers associated with lifestyle factors—including smoking, vaping, and exercise—and understanding how these behaviors may influence one another. Such biomarkers have clear potential applications in forensic science, particularly for inferring aspects of a perpetrator’s lifestyle from biological evidence at crime scenes, as demonstrated in previous work [18,20,32,74,89]. While several studies have focused specifically on biomarkers of smoking [18,20,21,22], no research to date has examined vaping-associated biomarkers for this forensic purpose. We acknowledge the limited number of vapers included in our cohort; however, the observed results suggest that this dataset may provide a valuable foundation for identifying preliminary biomarkers linked to vaping behavior.

An important interpretive limitation is that our gene annotations reflect genomic proximity rather than functional causality. CpGs and DMRs are annotated using the nearest gene based on UCSC RefGene (hg19), which facilitates reporting and literature contextualization. However, DNA methylation changes do not necessarily affect expression of the annotated gene. Methylation at distal enhancers, silencers, or other regulatory elements—particularly in intergenic regions—can regulate genes located hundreds of kilobases to megabases away through three-dimensional chromatin looping [53]. Of our 44 significant DMRs, 19 (43%) occur in intergenic regions, where functional targets are inherently ambiguous. Future studies integrating methylation data with transcriptomic profiles from matched buccal samples would be necessary to identify the actual target genes affected by these epigenetic changes. Additionally, tissue-specific enhancer maps from projects such as ENCODE or Roadmap Epigenomics could help prioritize candidate target genes for experimental validation.

## 5. Conclusions

This study evaluated DNA methylation changes in buccal cells because of smoking and vaping in a young adult population, finding potential new biomarkers of tobacco and e-cigarette consumption related to drug metabolism, oxidative stress, cell homeostasis, and immune response. Additionally, the impact of exercise on this population was assessed, including DNA methylation patterns changes because of sports performance, and identifying biomarkers among different groups.

This work has several limitations: (1) Age range and smoking duration: The cohort (18–31 years) was intentionally selected to capture early-stage smoking/vaping effects but limits detection of cumulative biomarkers like AHRR that require longer exposure. (2) Small sample sizes: Particularly in the vaper group (n = 12 total) and sports-stratified subgroups (n = 3–9), limiting statistical power for some comparisons. (3) Lack of intensity data: We did not collect quantitative smoking/vaping intensity (cigarettes/day, vaping frequency, pack-years), precluding dose–response analyses. Duration data were available (mean of 3.2 years for smokers, 2.1 years for vapers) but showed limited range. Future studies should incorporate standardized questionnaires capturing detailed exposure metrics (e.g., Fagerström Test for Nicotine Dependence, cotinine levels) to enable dose–response modeling. (4) Sports heterogeneity: Our “Sports” classification combined diverse activities (fencing, basketball, weightlifting, bowling) with varying intensities and energy expenditures, potentially diluting true effects of high-intensity athletic activity.

Future research aims to overcome these limitations, increasing the number of smokers and vapers, and expanding the age range to compare in the same cohort, methylation changes in long-term smokers with respect to recent smokers/vapers, to confirm these findings, and the effect of exercise. This could also lead to the discovery of new biomarkers for smoking and vaping status and exercise performance, with applications in clinical and forensic settings.

**Figure 5:**
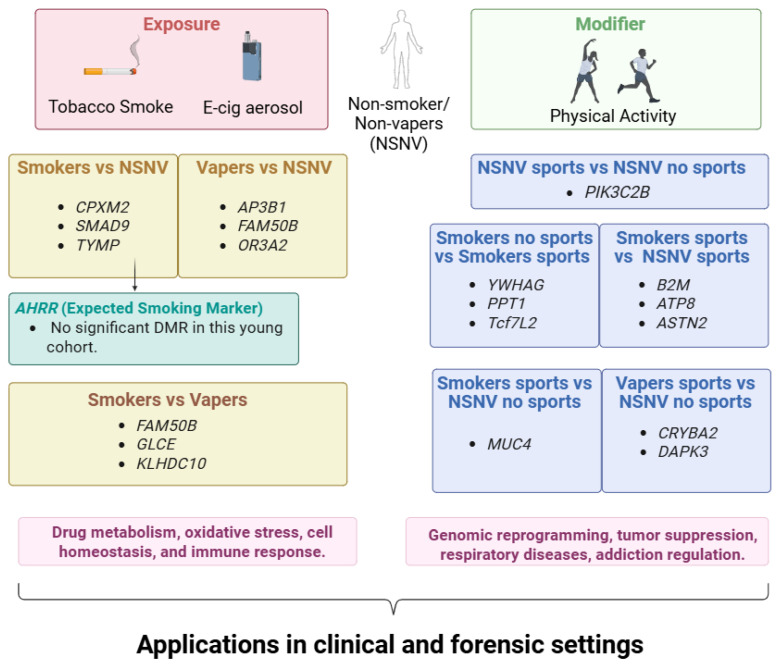
Epigenetic biomarkers associated with smoking, vaping, and physical activity. Selected loci discussed in the manuscript are shown; full results are available in the Supplementary Materials. Created with BioRender.com.

A summary of these findings is illustrated in Figure 5.

## Figures and Tables

**Table 1:** Summary of the participants in the study.

Condition	No Sports		Sports	
Biological Sex	Female	Male	Female	Male
Non-smoker/Non-vaper	31	18	35	46
Smoker	3	6	8	21
Vaper	2	1	4	5

Note: Fifteen participants reported both smoking and vaping; these dual users are classified as “Smoker” (smoking priority). The “Vaper” group represents exclusive e-cigarette users only. Duration data available for 35/38 smokers (mean of 3.2 ± 2.1 years) and 11/12 vapers (mean of 2.1 ± 2.3 years). Sex distribution by group: Smokers (38 total): 11 female/27 male; Vapers (12 total): 6 female/6 male; Non-smokers/Non-vapers (129 total): 66 female/64 male.

**Table 2:** DMR summary of the top differentially methylated genes in the three groups.

Comparison	Region	Gene(s)	Mean Methylation Difference (%)	FDR	#CpGs	Direction
Smokers vs. Non-smokers/non-vapers	chr10:123922970 -123923099	*CPXM2*	−4.53%	<0.001	5	Hypo in smokers
	chr22:50527953-50528641	*TYMP*	−2.39%	<0.001	13	Hypo in smokers
	chr13:36871646-36872346	*SMAD9*	−2.06%	<0.001	11	Hypo in smokers
Vapers vs. Non-smokers/non-vapers	chr5:78281103 -78282099	*AP3B1*	9.20%	<0.001	10	Hyper in vapers
	chr17:3289363-3290164	*OR3A2*	5.42%	<0.001	8	Hyper in vapers
	Chr6: 3848243-3849818	*FAM50B*	4.9%	<0.001	27	Hyper in vapers
Smokers vs. Vapers	chr15:69222400 -69223774	*GLCE*	−6.90%	<0.001	9	Hypo in smokers
	chr6:3848634-3849818	*FAM50B*	−5.49%	<0.001	25	Hypo in smokers
	chr7:130130740-130132453 (31 CpGs)	*KLHDC10*	−3.18%	<0.001	31	Hypo in smokers

## Data Availability

The data presented in this study are available from the corresponding author upon reasonable request.

## Supplementary Materials

The following supporting information can be downloaded at: https://www.mdpi.com/article/10.3390/genes17040369/s1. Supplementary Table S1: Participants’ lifestyle data. Supplementary Table S2: All differentially methylated regions (DMRs) from three primary comparisons (smokers vs. non-smokers/non-vapers; vapers vs. non-smokers/non-vapers; smokers vs. vapers) with full genomic annotations, rounded *p*-values, and comprehensive legend defining all columns and methods. Supplementary Table S3: Top CpG sites from sports-stratified comparisons (discovery threshold *p* < 1 × 10^−4^) with genomic annotations. Supplementary Table S4: *AHRR cg05575921* detailed analysis across all comparisons with explanation of non-significance. Supplementary Table S5: Sample classification table showing exclusive vs. dual users with demographics. Supplementary Table S6: Comparative summary table of key methylation findings across genes and groups for easy cross-comparison. Supplementary Figure S1: *AHRR cg05575921* methylation by smoking and sports status (boxplots and interaction plot). All genomic coordinates reference hg19 (GRCh37) genome build.
