# Supplementary figures and images for: The Epigenetic Fingerprint of Lifestyle: Smoking, Vaping, and Exercise Revealed Through Buccal DNA Methylation
Source: Genes (Basel). 2026 Mar 25;17(4):369. doi: 10.3390/genes17040369 (PMC13116420; doi:10.3390/genes17040369)

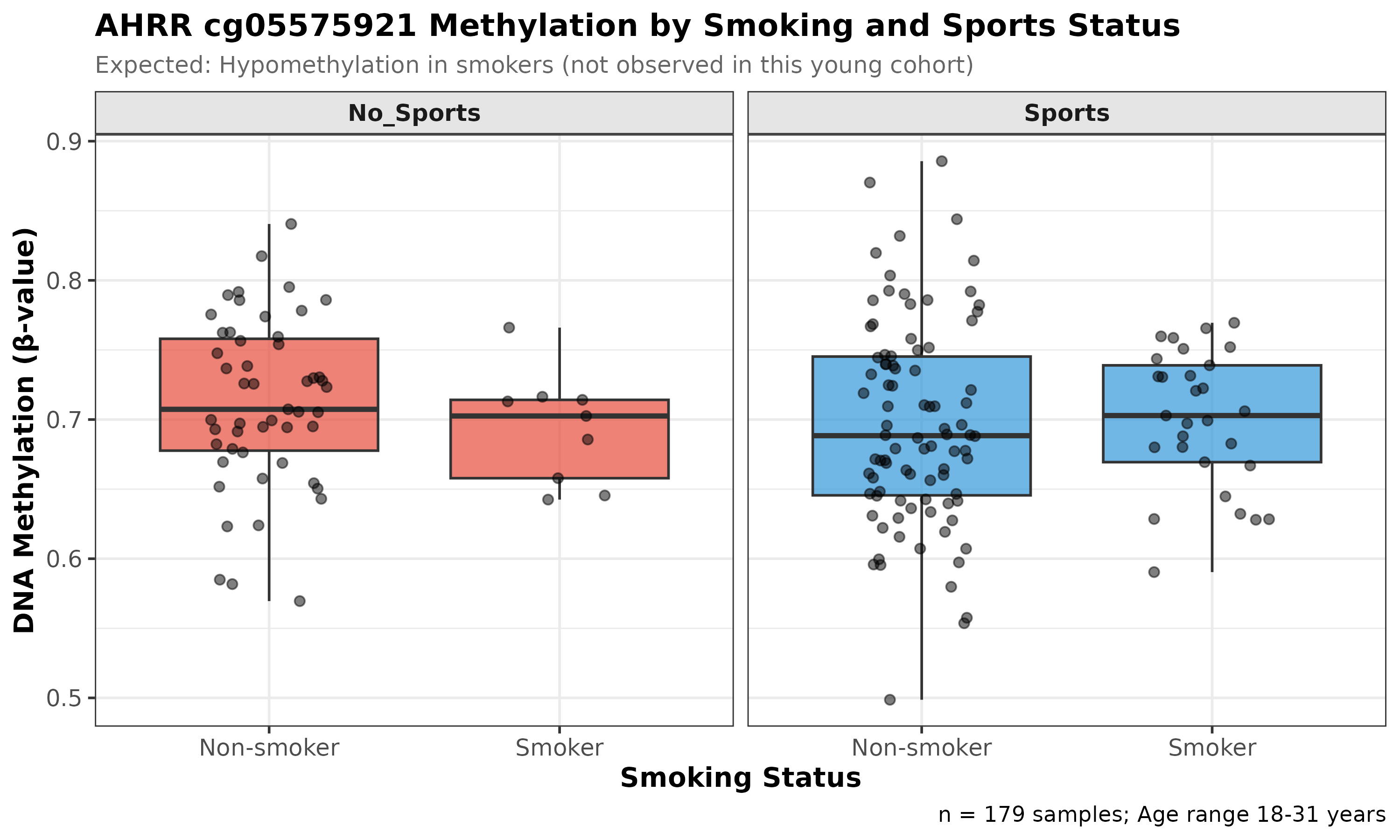

Supplement: Supplementary file 1 [file genes-17-00369-s001.zip › Supplementary_Figure_S1A_AHRR_Boxplot.png]

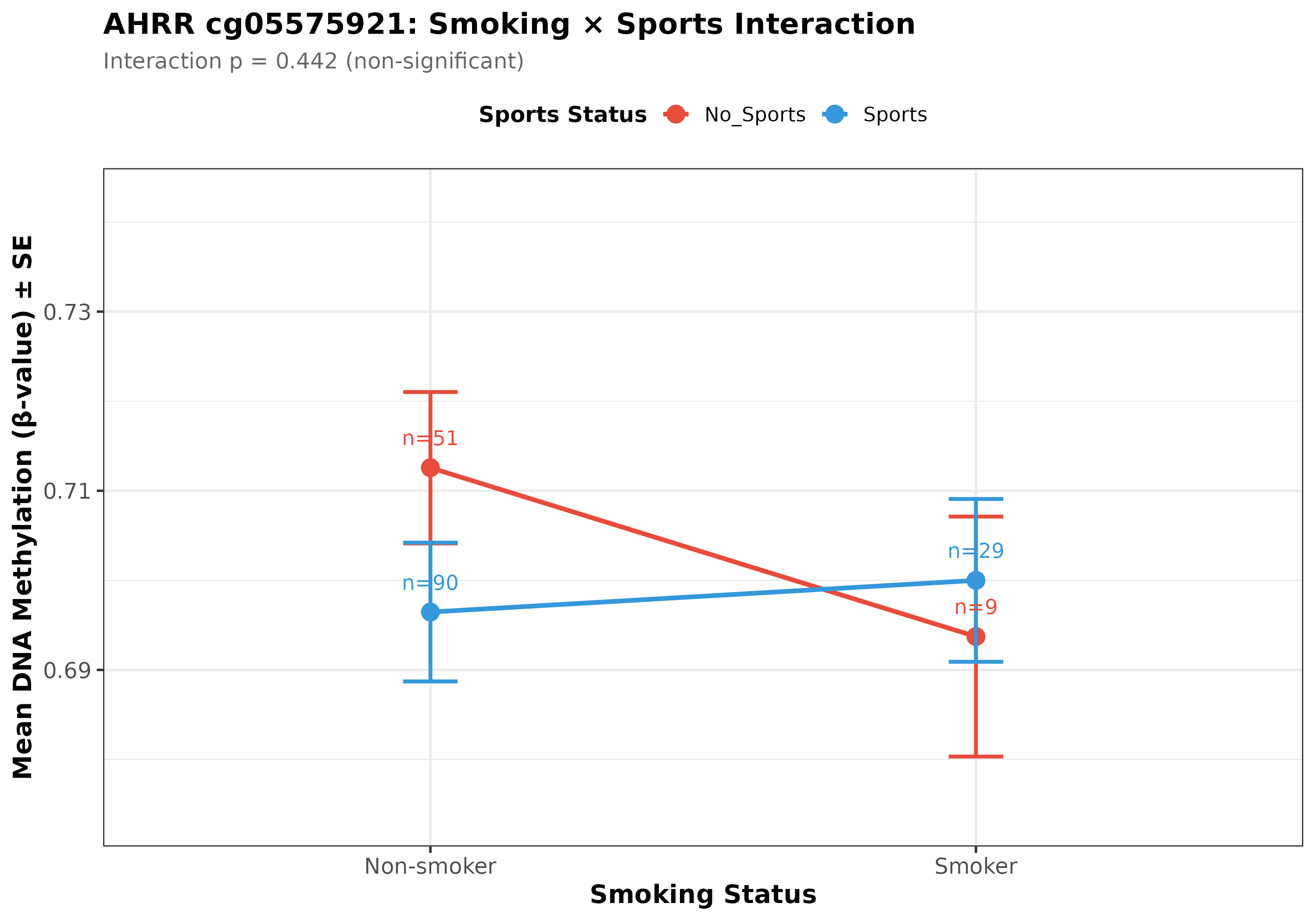

Supplement: Supplementary file 1 [file genes-17-00369-s001.zip › Supplementary_Figure_S1B_AHRR_Interaction.png]
